# Equatorial Spitting Cobra (*Naja sumatrana*) from Malaysia (Negeri Sembilan and Penang), Southern Thailand, and Sumatra: Comparative Venom Proteomics, Immunoreactivity and Cross-Neutralization by Antivenom

**DOI:** 10.3390/toxins14080522

**Published:** 2022-07-29

**Authors:** Choo Hock Tan, Kae Yi Tan, Kin Ying Wong, Nget Hong Tan, Ho Phin Chong

**Affiliations:** 1 Venom Research and Toxicology Laboratory, Department of Pharmacology, Faculty of Medicine, University of Malaya, Kuala Lumpur 50603, Malaysia; kinying12@gmail.com (K.Y.W.); schp_182@hotmail.com (H.P.C.); 2 Protein and Interactomics Laboratory, Department of Molecular Medicine, Faculty of Medicine, University of Malaya, Kuala Lumpur 50603, Malaysia; kytan_kae@um.edu.my (K.Y.T.); tanngethong@yahoo.com.sg (N.H.T.)

**Keywords:** venom variation, antivenom potency, *Naja kaouthia* monovalent antivenom (NkMAV), monocled cobra antivenom, snakebite envenoming

## Abstract

The Equatorial Spitting Cobra (*Naja sumatrana*) is a medically important venomous snake species in Southeast Asia. Its wide geographical distribution implies potential intra-specific venom variation, while there is no species-specific antivenom available to treat its envenoming. Applying a protein-decomplexing proteomic approach, the study showed that three-finger toxins (3FTX), followed by phospholipases A_2_ (PLA_2_), were the major proteins well-conserved across *N. sumatrana* venoms of different locales. Variations were noted in the subtypes and relative abundances of venom proteins. Of note, alpha-neurotoxins (belonging to 3FTX) are the least in the Penang specimen (Ns-PG, 5.41% of total venom proteins), compared with geographical specimens from Negeri Sembilan (Ns-NS, 14.84%), southern Thailand (Ns-TH, 16.05%) and Sumatra (Ns-SU, 10.81%). The alpha-neurotoxin abundance, in general, correlates with the venom’s lethal potency. The Thai *Naja kaouthia* Monovalent Antivenom (NkMAV) was found to be immunoreactive toward the *N. sumatrana* venoms and is capable of cross-neutralizing *N. sumatrana* venom lethality to varying degrees (potency = 0.49–0.92 mg/mL, interpreted as the amount of venom completely neutralized per milliliter of antivenom). The potency was lowest against NS-SU venom, implying variable antigenicity of its lethal alpha-neurotoxins. Together, the findings suggest the para-specific and geographical utility of NkMAV as treatment for *N. sumatrana* envenoming in Southeast Asia.

## 1. Introduction

Removed from the list of Neglected Tropical Diseases (NTD) in 2013, snakebite envenoming was finally redesignated as a priority neglected tropical disease by the World Health Organization (WHO) in 2017 [1,2]. Yearly, an estimate of 81,000–138,000 deaths and approximately 400,000 disabilities are resultant from this global scourge worldwide [1,3]. The problem disproportionately affects impoverished rural populations in many developing countries, including those in Southeast Asia where venomous snakes such as cobras are abundant and commonly implicated in snakebite envenoming [4,5].

Cobras play significant roles in many popular cultures and are well known for their medical and public health importance. Specifically, “true cobras” are members of the genus *Naja*, which contains various species occurring in regions throughout Africa, Southwest Asia, South Asia, Southeast Asia and East Asia [6]. In Southeast Asia, there are at least six to seven distinct species of cobras that are able to inflict fatal and disabling envenomation [4,7]. Of these, the Equatorial Spitting Cobra, *Naja sumatrana*, is distributed throughout the Malay Peninsula (including the islands of Penang and Singapore), Sumatra, the southern part of Thailand, and part of Borneo Island. The species was previously grouped under a larger species complex of *Naja naja sputatrix*, locally known as theMalayan Spitting Cobra without a clear distinction from other spitting cobras in the region, which are now elevated to different species such as *Naja siamensis* in Indochina, and *Naja sputatrix* on the Java Island of Indonesia [8]. To note, the *N. sumatrana* species is morphologically varied, especially in body coloration, with a transition from yellow or light brown in southern Thailand, northern West Malaysia and Penang Island to black in most parts of the Malay Peninsula (south to Kedah), Singapore, Sumatra, and Borneo. As cobras are diversely distributed in the region, the diagnosis of species is crucial for proper snakebite management, improved prognosis, and in fatality cases for forensic inputs [9].

Clinically, the majority of cobra species produce neurotoxic and cytotoxic envenoming syndrome, attributed to the effects of neurotoxins and cytotoxins present in their venoms [7]. Nevertheless, snake venoms can vary substantially between species in terms of protein composition and antigenicity, thus resulting in variable toxic manifestations and treatment effectiveness [1,10,11,12]. Consequently, the identification of cobra species is critical in the management of snakebite envenoming. Venom variability has also been widely recognized within the same snake species, in particular those with a wide biogeographical distribution, as distant populations evolved variable venoms for adaptation to the distinct ecological niche. For instance, in Southeast Asia, marked geographical venom variation has been reported for the Monocled Cobra (*Naja kaouthia*) from Malaysia, Thailand and Vietnam, showing variable venom proteomes that correlate with venom toxicity and discrepancy in the neutralization efficacy of antivenom [13,14]. Similarly, intra-specific variations in snake venom composition, antigenicity and neutralization have been reported for various other medically important snakes in Southeast Asia, including Russell’s Viper, Malayan Pit Viper, Malayan Krait, Banded Krait and King Cobra [15,16,17,18,19,20]. The variations have ramifications on antivenom use, as antivenoms are usually domestic products manufactured in one locale and thus may have limited efficacy in neutralizing the venom of the same snake species from another geographical area. Moreover, in Southeast Asia, the antivenom products have limited species coverage, leaving most snakes in the region without specific antivenom. Consequently, non-specific antivenoms are commonly used as para-specific treatment for envenoming caused by many snake species in this region [7,21]. In this regard, the Thai *Naja kaouthia* Monovalent Antivenom (NKMAV) is the recommended treatment for *N. sumatrana* envenoming, given that Neuro Polyvalent Antivenom, a poly-specific antivenom raised against the venoms of *N. kaouthia*, *Bungarus candidus*, *Bungarus flaviceps* and *Ophiophagus hannah*, has been found to be effective in cross-neutralizing *N. sumatrana* venom sourced from Seremban in West Malaysia [22,23]. Nevertheless, the potential geographical variability of *N. sumatrana* venom and its impact on the immunoreactivity and neutralization activity of antivenom have not been investigated. 

This study therefore aimed to explore the venom variation of *N. sumatrana* sourced from four geographical locales, i.e., southern Thailand, Peninsular Malaysia (Negeri Sembilan), Penang Island (West Malaysia), and the island of Sumatra (Indonesia), applying a decomplexation proteomic approach for snake venom [24]. Furthermore, the immunoreactivity and neutralization efficacy of NkMAV, which is the clinically recommended monovalent antivenom for treatment, was assessed against the different venoms using an in vitro immunoassay and an in vivo murine model. The findings shall provide deeper insights into the intra-specific venom variability of *N. sumatrana* and valuable information for antivenom use optimization in the region. 

## 2. Results

### 2.1. Chromatographic and Electrophoretic Profiles of Naja sumatrana Venoms 

**Figure 1:**
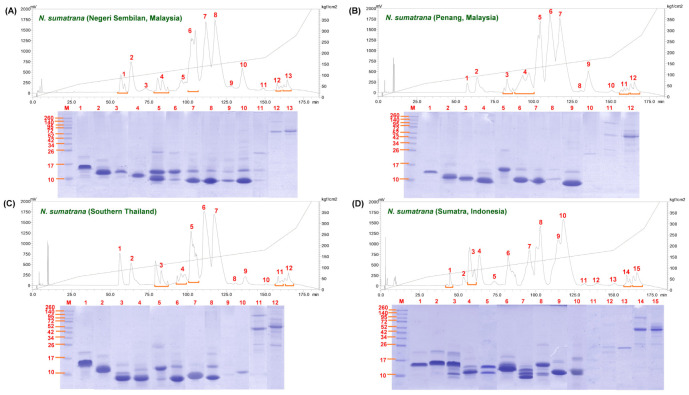
Protein decomplexation profiles of *Naja sumatrana* venoms sourced from (**A**) Negeri Sembilan, Malaysia, (**B**) Penang, Malaysia, (**C**) Southern Thailand, and (**D**) Sumatra, Indonesia. Upper panel: chromatographic profiles of venoms by C18 reverse-phase high-performance liquid chromatography. Lower panel: electrophoretic profiles of venom proteins separated by 15% sodium dodecyl sulfate-polyacrylamide gel under reducing conditions.

C18 reverse-phase HPLC resolved *N. sumatrana* venoms into 12 to 15 fractions over a time course of 180 min (Figure 1). The venoms of *N. sumatrana* from Negeri Sembilan (Ns-NS), Penang (Ns-PG) and southern Thailand (Ns-TH) exhibited similar elution patterns based on the chromatograms, whereas that from Sumatra (Ns-SU) showed notable differences between 100 and 125 min, a region where the majority of proteins were eluted for the four venoms. 

Electrophoretic profiles of the chromatographic fractions of all four venoms showed the proteins eluted from 55 to 140 min were of low molecular weight (MW) (<15 kDa), accounting for >92% of the total venom proteins based on the AUC (area under the curve) of the respective venoms. Minor proteins were eluted in the later course of the fractionation, where heterogeneous mixtures of low and moderate MW proteins (15–30 kDa) were eluted between 140 and 150 min, while high MW proteins (>40 kDa) eluted between 155 and 170 min (Figure 1). 

### 2.2. Naja sumatrana Venom Proteomes

The identification and quantitation of *N. sumatrana* venom proteins fractionated by RP-HPLC are shown in Table 1. Mass spectrometric data including parameters for spectral ions, protein scores, and amino acid sequences of peptides are provided in Supplementary Materials.

**Figure 2:**
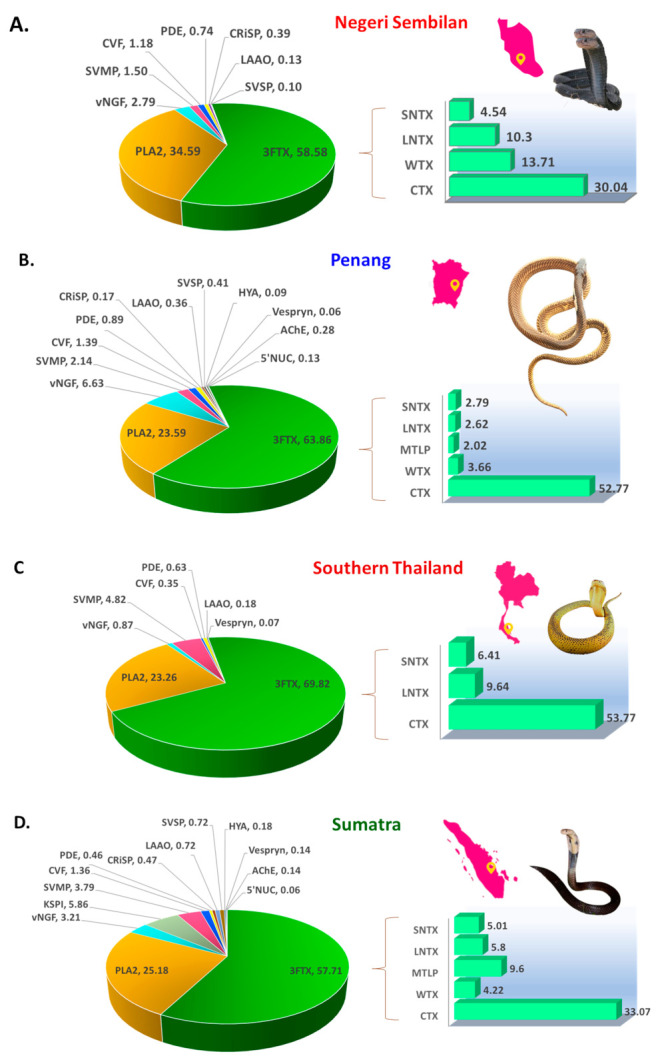
Venom proteomes of *Naja sumatrana* profiled using C18 RP-HPLC followed by tandem mass spectrometry for specimens from various geographical locales: (**A**) Negeri Sembilan, Malaysia (Ns-NS), (**B**) Penang, Malaysia (Ns-PG), (**C**) Southern Thailand (Ns-TH), and (**D**) Sumatra, Indonesia (Ns-SU). Abbreviation: 3FTx, three-finger toxin; PLA_2_, phospholipase A_2_; vNGF, venom nerve growth factor; KSPI, Kunitz-type serine protease; SVMP, snake venom metalloproteinase; CVF, cobra venom factor; PDE, phosphodiesterase; CRiSP, cysteine-rich secretory protein; LAAO, L-amino acid oxidase; SVSP, snake venom serine protease; HYA, Hyaluronidase; AChE, acetylcholinesterase; and 5′NUC, 5′nucleotidase. Bar graphs represent the sub-proteome of three-finger toxins in the venoms. Insets: Images of *N. sumatrana* with adaptations: A, www.reptilefact.com/wp-content/uploads/2016/08/Equatorial-Spitting-Cobras.jpg, accessed on 21 July 2022; B, author CHT; C, Joel Sartore; D, Gernot Vogel (www.reptile-database.reptarium.cz/species?genus=Naja&species=sumatrana, accessed on 21 July 2022).

In comparison, a total of 22–55 proteoforms, belonging to 8–14 protein families, were identified in *N. sumatrana* venoms at varying abundances (Table 1). Most proteins were annotated by matching homologous toxin sequences to *Naja* species available in the databases used for data mining. Among the protein families identified, three-finger toxins (3FTX), phospholipase A_2_ (PLA_2_), venom nerve growth factor (vNGF), snake venom metalloproteinase (SVMP), cobra venom factor (CVF), phosphodiesterase (PDE) and L-amino acid oxidase (LAAO) are the seven protein families consistently detected in all four *N. sumatrana* venoms. Cysteine-rich secretory protein (CRiSP) and snake venom serine protease (SVSP) were detected in all *N. sumatrana* venoms, except for Ns-TH, whereas vespryn was detected in all samples, except for Ns-NS. Hyaluronidase (HYA), acetylcholinesterase (AChE) and 5′ nucleotidase (5′NUC) were only detected in Ns-PG and Ns-SU, while Kunitz-type serine protease (KSPI) was distinctively present in Ns-SU. The overall proteomic profiles of the four venoms are illustrated in Figure 2.

Across the venom proteomes of *N. sumatrana* from all four locales, 3FTX constitutes the most abundant protein family, accounting for 57–70% of the total venom proteins. The 3FTX proteins include subtypes of long neurotoxins (LNTX, 2.6–10.3%), short neurotoxins (SNTX, 2.8–6.4%), cytotoxin (CTX, 30.0–54.0%), muscarinic toxin-like protein (MTLP, 2.0–9.6%) and weak toxins (WTX, 3.7–13.7%) (Table 1; Figure 2). LNTX, SNTX and CTX were present in all four venoms, while MTLP was detected in Ns-PG and Ns-SU venoms, and WTX was detected in Ns-NS, Ns-PG and Ns-SU venoms. The second most abundant protein of all four venoms was PLA_2_, which constitutes 23.3–34.6% of total venom proteins. Toxins belonging to the remaining protein families have lower abundances, each accounting for less than 7% of the total venom proteins. These include vNGF (0.9–6.6%), KSPI (5.9%, solely detected in Ns-SU), SVMP (1.5–4.8%), CVF (0.4–1.4%), PDE (0.5–0.9%), CRiSP (0.2–0.5%), LAAO (0.1–0.7%), SVSP (0.1–0.7%), HYA (0.1–0.2%), AChE (0.1–0.3%) Vespryn (~0.1%) and 5′NUC (~0.1%) (Figure 2).

### 2.3. Immunoreactivity of Naja kaouthia Monovalent Antivenom (NkMAV)

**Figure 3:**
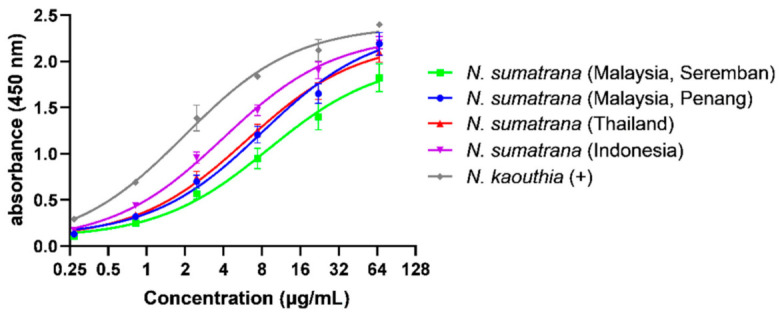
Concentration-dependent immunological binding activity of *Naja kaouthia* Monovalent Antivenom (NkMAV) toward *Naja sumatrana* venoms from four different geographical locales. *Naja kaouthia* venom was used as positive control. Values were means ± S.E.M of triplicates.

In indirect ELISA, the immunoreactivity of NkMAV increased dose dependently toward the venoms tested (Figure 3). NkMAV has high immunoreactivity toward the *N. kaouthia* venom, which served as the positive control, with a half-maximal effective concentration (EC_50_) of 2.04 ± 0.32 µg/mL and a maximal absorbance (Ab_max_) of 2.40 ± 0.03. In comparison, its immunoreactivity significantly reduced when testing against the *N. sumatrana* venoms and varied among these venoms (EC_50_ = 4.06–9.64 µg/mL, Ab_max_ = 1.82–2.20) (Table 2).

### 2.4. Venom Lethality and Neutralization by NkMAV

The four samples of *N. sumatrana* venom were lethal to mice. Ns-SU, Ns-TH and Ns-NS have median lethal doses (LD_50_) ranging between 0.39 and 0.50 µg/g. Ns-SU was the most potent (LD_50_ = 0.39 µg/g), comparable to the venom of Ns-TH (LD_50_ = 0.41 µg/g) and Ns-MS (LD_50_ = 0.50 µg/g) (Table 3). In comparison, Ns-PG has a higher LD_50_ value (0.96 µg/g) and is almost one-fold less lethal than the three other *N. sumatrana* venoms.

In the neutralization experiment, NkMAV effectively neutralized the lethality of all *N. sumatrana* venoms, with values of normalized potency (n-P) ranging from 11–21 mg/g (milligram of venom completely neutralized per gram of antivenom protein). Based on the previous study, NkMAV neutralization activity against the homologous *N. kaouthia* venom was comparable, with an n-P value of 20.44 mg/g. In the present study, the neutralization effects of NkMAV against the Ns-NS, Ns-PG and Ns-TH venoms were similar (n-P~20 mg/g), whereas the potency against Ns-SU was approximately one-fold lower (n-P = 11.4 mg/g) (Table 3). 

## 3. Discussion

The decomplexation proteomic approach allows for the comparison of venom compositions of *N. sumatrana* from different geographical locales. 3FTX proteins dominated the venom proteomes, and within 3FTX are cobra-characteristic toxins, i.e., short-chain and long-chain alpha-neurotoxins (SNTX and LNTX), and cytotoxins (CTX), well conserved across the different geographical specimens. The alpha-neurotoxins block post-synaptic nicotinic acetylcholine receptors (nAChR) at the neuromuscular junctions, thereby inhibiting the neurotransmission and causing paralysis, which is the primary mode of death in cobra envenoming [26,27]. The alpha-neurotoxin abundance (quantitatively expressed in percentage of total venom proteins) correlates with the lethal potency of cobra venom, and these toxins therefore should be the main target for immunological binding and neutralization by antivenom [28,29]. Earlier, the venom proteome of *N. sumatrana* sourced from Seremban (Malaysia) showed an alpha-neurotoxin abundance of 15% [30], a value comparable to our current finding of 14.84% and 16.05% alpha-neurotoxins for *N. sumatrana* from Negeri Sembilan (Malaysia) (Ns-NS) and southern Thailand (Ns-TH), respectively. The present study further unveils the variable alpha-neurotoxin abundances of *N. sumatrana* from Penang (Ns-PG) and Sumatra (Ns-SU). Of note, the venoms of both insular origins contained less alpha-neurotoxins compared with the continental venoms, where Ns-PG has the lowest abundance (5.41%) and accordingly, a lower lethal potency (discussed below). 

While SNTX and LNTX are present across all *N. sumatrana* venoms tested, the proteoforms and relative abundances varied among the venoms. The differences might influence the venom toxicity, antigenicity and neutralization, thus resulting in variable clinical manifestation and response to antivenom treatment. Previous studies indicated that SNTX, in contrast to LNTX, binds less irreversibly to nAChR and is therefore, theoretically, less medically relevant to envenoming in humans [31,32]. Nevertheless, SNTX still contributes to the overall venom lethality, especially for cobras of eastern dispersal, e.g., *Naja sputatrix* and *Naja atra* whose alpha-neurotoxins are predominantly SNTX [33,34], and *Naja philippinensis* as well as *Naja samarensis*, whose alpha-neurotoxins are exclusively SNTX [28,35]. In addition, previous studies showed that commercial antivenoms have a lower neutralization potency against SNTX than LNTX, suggesting SNTX is the limiting factor of antivenom efficacy, presumably because of its small molecular size and fewer epitopes, accompanied with a low antigenicity [25,36,37,38]. Thus, the ratio between the two alpha-neurotoxins could influence the efficacy of a heterologous antivenom, such that cobra venoms rich in SNTX might not be well neutralized by an antivenom raised against LNTX-dominant cobra venoms. The present study provides the relative abundances of SNTX and LNTX, and shows that *N. sumatrana* venoms from Negeri Sembilan (Ns-NS) and southern Thailand (Ns-TH) contained slightly more LNTX than SNTX (ratios of LNTX:SNTX~1.5–2:1). Conversely, *N. sumatrana* from Penang (Ns-PG) and Sumatra (Ns-SU), representing insular populations, have a more balanced expression ratio of LNTX and SNTX in the venom (LNTX:SNTX = 1:1). The antivenom proposed for cross-neutralization of *N. sumatrana* venom, i.e., NkMAV, is raised against the Thai Monocled Cobra venom, which contains abundant LNTX (as its dominant form of alpha-neurotoxins) and relatively less SNTX (33.3% LNTX, 7.7% SNTX) [14]. In comparison, the *N. sumatrana* venoms have lower LNTX abundances (2.62–10.3%) and a considerably balanced distribution of LNTX and SNTX. The amino acid sequences of LNTX of *N. kaouthia* and *N. sumatrana*, despite variation, demonstrate high homology between the two species, and the same is true for their SNTX [39,40]. Accordingly, NkMAV was able to cross-neutralize the *N. sumatrana* venoms of different locales, albeit with varying degrees of potency (discussed below). A recent bioinformatic study also suggested that SNTX and LNTX, despite their small molecular sizes and toxicities, are still immunogenic but effective adjuvants, and improved immunization procedures are needed for the production of potent antivenoms against these elapid neurotoxins [41].

Cobra cytotoxins (CTX), conventionally called cardiotoxins, are universally present in cobra venoms across all African and Asiatic lineages of *Naja* spp. [42,43,44,45,46,47,48]. These are toxins mainly implicated in the pathogenesis of cytotoxicity and tissue necrosis [49,50,51,52], and they are lethal when administered intravenously in mice. CTX have, however, much higher LD_50_ (>1 µg/g, implying lower lethality) compared with the highly lethal alpha-neurotoxins (LD_50_ < 0.2 µg/g). The present study shows that CTX constitutes the main bulk of *N. sumatrana* venom proteins (~30–60%), and their high abundance supports the occurrence of extensive tissue necrosis associated with local envenoming by *N. sumatrana* [5,7,53]. A recent study showed that the local cytotoxic effect caused by *N. sumatrana* venom and its CTX could be cross-neutralized by commercially available *N. kaouthia* and *Naja atra* antivenoms, although the efficacy was generally low, a finding consistent with limited antivenom effectiveness in the clinical treatment of local tissue damage caused by cobra bites [49]. Other 3FTX detected in *N. sumatrana* venom proteomes are different forms of weak toxins and muscarinic toxin-like proteins, whose roles in the clinical pathophysiology of envenoming remain obscure. 

Snake venom PLA_2_ are secretory enzymatic proteins with diverse pharmacological activities, and they are commonly present in snake venoms [53]. Following 3FTX, PLA_2_ made up the second most abundant protein family in all *N. sumatrana* venoms, a phenotypic pattern similarly observed across various cobra species including *N. sumatrana* from Seremban [29,43,44,46,48]. Cobra venom PLA_2_ follows a unique distribution pattern across different *Naja* subgenera. PLA_2_ content is absent or negligible in the venoms of African non-spitting cobras (subgenus *Uraeus*) but markedly higher in the other subgenera (*Naja*, *Afronaja* and *Boulengerina*) especially spitting cobras, presumably associated with the venom algesic (pain-inducing) properties for defense [51,54]. The PLA_2_ forms identified in *N. sumatrana* venom proteomes are diverse. The *N. sumatrana* venoms contain acidic PLA_2_ which are present virtually in all cobra venoms, and the acidic PLA_2_ generally lack lethal activity in mice [25,36]. In addition, the *N. sumatrana* venoms also contain highly conserved neutral forms of PLA_2_, which have been shown to be lethal to mice [23]. Nonetheless, the neutral PLA_2_ of *N. sumatrana*, with an intravenous LD_50_ of 2 µg/g in mice, is less lethal than the toxic PLA_2_ of *N. sputatrix* (LD_50_ = 0.5 µg/g, near-neutral PLA_2_) [34] and *Hydrophis schistosus* (Beaked Sea Snake) (LD_50_ = 0.08 µg/g, basic PLA_2_) [55]. Comparing between the present study and the earlier report for *N. sumatrana* from Seremban, the relative abundance of PLA_2_ ranged between 23% and 34% of total venom proteins in the current work, whereas the PLA_2_ abundance was only 7.2% as reported previously [30]. In addition, while LCMS/MS analysis in the present work detected 4–6 PLA_2_ proteoforms, two were reported by the previous study. The relative abundance of PLA_2_ in *N. sumatrana* venoms (present study) is comparable with that reported for *N. sputatrix* venom [34], in line with the anticipated high PLA_2_ abundance for spitting cobras. Nonetheless, there are exceptions in two Asiatic spitting cobras (*N. philippinensis* and *N. samarensis*), who have greatly downregulated the expression of PLA_2_ and CTX while conserving the venom-spitting trait [28,35]. 

Other minor proteins detected in *N. sumatrana* venoms are vNGF, SVMP, CVF, PDE, CRiSP, LAAO, SVSP, HYA, AChE, Vespryn, 5′NUC and KSPI. These are, in general, proteins with high molecular weights (contrary to the major toxin group, 3FTX), and are less abundant in the venoms, suggesting ancillary functions without direct lethal implication. Certain toxins are known to play roles in inflammatory responses (e.g., SVMP, CRiSP) and facilitate venom dissemination (e.g., CVF, PDE, 5′NUC) during snakebite envenoming [56,57,58,59].

In mice, Ns-NS, Ns-TH and Ns-SU venoms showed comparable lethality (LD_50_ = 0.4–0.5 µg/g), while Ns-PG venom was significantly less lethal (LD_50_ = 0.9 µg/g). In cobra envenoming, lethality is primarily a sequel of neuromuscular paralysis caused by the alpha-neurotoxins, whose protein abundance positively correlates with the venom’s lethal potency. The lethality of Ns-NS, Ns-TH and Ns-SU venoms is considered moderate in comparison to the Thai *N. kaouthia*, Pakistani *N. naja*, Philippine’s *N. philippinensis* and *N. samarensis* venoms, which have much higher abundances of alpha-neurotoxins (23–66%) and are thus more potent for lethal effect (LD_50_ = 0.1–0.2 µg/g) [14,35,60]. In contrast, Ns-PG venom, with the least alpha-neurotoxins (~5% of total venom proteins) among the four *N. sumatrana* venoms, is less neurotoxic and less lethal. This is a venom characteristic shared by the Javan *N. sputatrix*, Malaysian and Vietnamese *N. kaouthia*, and Taiwanese *N. atra* which are generally considered less neurotoxic (LD_50_ ≥ 0.9 µg/g) [14,33,48,60]. 

Presently, there is no species-specific antivenom available against *N. sumatrana*, and thus, hetero-specific antivenom products are recommended by the Clinical Guideline of Malaysia for treatment of *N. sumatrana* envenoming [21]. As both species of *N. kaouthia* and *N. sumatrana* share close geographical proximity, NkMAV (raised against the Thai *N. kaouthia* venom) as a monovalent antivenom commercially available in Southeast Asia appears to be the appropriate choice of antivenom for *N. sumatrana* envenoming. The ELISA finding showed that NkMAV is immunoreactive toward all *N. sumatrana* venoms, suggesting largely conserved venom antigenicity between *N. kaouthia* and *N. sumatrana*, and among specimens of the latter from different geographical locales. The significantly lower immunoreactivity of NkMAV toward *N. sumatrana* venoms (in comparison to *N. kaouthia* venom), however, implies subtle antigenic divergence between *N. kaouthia* and *N. sumatrana* venom proteins. Among the *N. sumatrana* venoms, NkMAV immunoreactivity varied with the highest activity noted in Ns-SU, followed by Ns-TH, Ns-PG and Ns-NS. The immunoreactivity of NkMAV reflects the general binding activity of antibody toward various protein antigens, including lethal and non-lethal components in the venoms. Hence, while it is a good indicator of antibody–antigen (toxin) binding, it does not necessarily provide accurate prediction for antivenom neutralization efficacy. The cross-reactivity shown, nonetheless, suggests potential cross-neutralization, which should be subsequently evaluated with functional toxicity assays, such as in vivo neutralization of lethality which is the gold standard as per the WHO guideline [60,61]. 

Thus, using a mouse model, this study further confirmed the in vivo efficacy of NkMAV in cross-neutralizing the lethal effect of *N. sumatrana* venoms. Its neutralization activity for Ns-TH, Ns-PG and Ns-NS is moderate, with a normalized potency of approximately 20 mg/g (interpreted as the amount of venom neutralized completely by one gram of antivenom proteins), which is comparable to its neutralization potency against the homologous Thai *N. kaouthia* venom (normalized potency = 20.44 mg/g) [25]. The neutralization against Ns-SU venom, however, is apparently weaker (by almost one-fold), indicating that a higher dose of antivenom may be needed to effectively cross-neutralize the lethal alpha-neurotoxins of Ns-SU. Presumably, the alpha-neurotoxins specific to the Indonesian *N. sumatrana* are antigenically more varied from *N. kaouthia*, and *N. sumatrana* of Malaysia (Ns-NS, Ns-PG) and Thailand (Ns-TH). Additionally, Ns-SU venom might possess synergistic toxins that are less neutralized by the antivenom NkMAV. The discrepancy unveiled should also be taken into consideration when formulating a “diverse toxin repertoire” immunogen for the development of a pan-specific, pan-regional antivenom product [62,63].

To put things into perspective, we estimate that *N. sumatrana* venom has an intravenous LD_50_ of approximately 0.04–0.07 mg/kg in humans, through converting the murine LD_50_ to human equivalent dose (multiplying the animal dose by 0.081) [64]. Accordingly, for an adult man weighing 60 kg, a few milligrams of the venom (>2.4–4.2 mg) with access to the circulation would be lethal. A full-grown cobra is capable of injecting copious venom, easily at any amount between 20–50 mg or more (author’s experience). Assuming 30 mg of injected venom is well absorbed during envenoming, and based on the neutralization potency of NkMAV, a dose consisting of 3–4 vials of antivenom would be needed for complete neutralization against the Ns-TH, NS-PG and Ns-NS venoms, while a higher dose (six vials) might be required for neutralizing the Ns-SU venom. In the absence of species-specific antivenom, this preclinical finding thus provides a guide to justify the para-specific use and the dosing of NkMAV for *N. sumatrana* envenoming. This is also consistent with clinical guidelines that recommend an initial dose of 100 mL (10 vials) of cobra antivenoms [4,21]. Although 10 vials of antivenom appears to be a high dose, it may be reasonable in anticipation of excessive venom injection and concurrent clearance of antivenom from the body. Furthermore, repeated antivenom doses will be needed in recurrent neurotoxic envenoming [65], where venom deposited at the bite site gradually enters the circulation while the antivenom effect wears off with time—a phenomenon described as the pharmacokinetic and pharmacodynamic mismatch between venom and antivenom. Hence, while the patient shows favorable response to initial antivenom treatment, recurrent syndrome must be closely monitored for prompt antivenom re-treatment.

## 4. Conclusions

This study shows intra-specific venom variation of *N. sumatrana* from four geographical locales in Southeast Asia. 3FTX constitutes the major protein family that is well conserved in the venoms, notwithstanding variable subtypes and abundances of the lethal alpha-neurotoxins within this protein family. Of note, the alpha-neurotoxin abundance is the lowest in Ns-PG venom, and this correlates with its significantly lower lethality among the venom specimens. The hetero-specific antivenom raised against Thai Monocled Cobra venom, NkMAV, shows dose-dependently increasing immunoreactivity toward the *N. sumatrana* venoms, suggesting conserved venom protein antigenicity between *N. kaouthia* and *N. sumatrana*. NkMAV was efficacious in cross-neutralizing the lethal effects of *N. sumatrana* venoms to varying degrees, with the lowest potency found against the Indonesian *N. sumatrana* venom, implying distinct antigenicity of its lethal neurotoxins. On the whole, the findings support NkMAV utility as a para-specific antivenom treatment for *N. sumatrana* envenoming in Southeast Asia. The antivenom dosage for cross-neutralization derived from the study suggests dosing consistent with most guidelines’ recommendations for *N. kaouthia* envenoming. Further study should aim to improve the antivenom potency through approaches such as improving the antibody purity, increasing the antivenom concentration, or incorporating toxin-specific antibodies to overcome the need for high doses of antivenom. 

## 5. Materials and Methods

### 5.1. Antivenoms and Venoms

The venoms of Malaysian *N. sumatrana* were sourced from Negeri Sembilan, Malaysia (Ns-NS) and Penang Island (Ns-PG). The venom of Indonesian *N. sumatrana* (Ns-SU) was obtained from local snake catchers from Sumatra, Indonesia, and the Thai *N. sumatrana* (Ns-TH) was supplied by Queen Saovabha Memorial Institute, Bangkok, Thailand. All venoms were obtained from adult specimens (n > 5), lyophilized and stored at −20 °C until use. The antivenom used was *Naja kaouthia* Monovalent Antivenom (NkMAV) manufactured by Queen Saovabha Memorial Institute (batch no.: NK00514) supplied in lyophilized form. NkMAV contained purified F(ab)’_2_ derived from sera of horses hyperimmunized against Thai *N. kaouthia* (Monocled Cobra) venom. The antivenom was reconstituted in 10 mL of distilled water prior to use.

### 5.2. Animal Supply and Ethics Statement

Mice used in this study were of albino ICR strain (20–25 g) supplied by the Animal Experimental Unit from the University of Malaya. The protocol for animal experimentation was carried out based on the Council for International Organizations of Medical Sciences (CIOMS) guidelines and was approved by the Institutional Animal Care and Use Committee of the University of Malaya (Ethics clearance number: 2014-09-11/PHAR/R/TCH).

### 5.3. Reverse-Phase High-Performance Liquid Chromatography (RP-HPLC)

Three milligrams of *N. sumatrana* venoms were reconstituted in 200 µL ultrapure water and the supernatant was subjected to C18 RP-HPLC. The LiChrospher^®^ WP 300 C18 column (5 µm particle size) column (Merck, Darmstadt, Germany) was pre-equilibrated with 0.1% TFA in water (Eluent A), and the sample was eluted with 0.1% TFA in acetonitrile (Eluent B) using linear gradients of 5% B for 10 min, 5–15% B over 20 min, 15–45% B over 120 min and 45–70% B over 20 min. The flow rate was set at 1 mL/min. Absorbance was monitored at 215 nm, and the eluted proteins indicated by resulting peaks were collected, lyophilized and kept at −20 °C prior to use.

### 5.4. Sodium Dodecyl Sulfate-Polyacrylamide Gel Electrophoresis (SDS-PAGE)

The fractions collected from RP-HPLC were reconstituted in ultrapure water and separated by 15% sodium dodecyl sulfate-polyacrylamide gel electrophoresis (SDS-PAGE) under reducing condition at 100 V for 2 h. Thermo Scientific Spectra™ Multicolor Broad Range Protein Ladder containing 10 prestained proteins ranging from 10 to 260 kDa was used for molecular mass calibration. Gel was stained with Coomassie Brilliant Blue R-250, and the bands were scanned using ImageScanner III (GE Healthcare, Uppsala, Sweden).

### 5.5. In-Solution Tryptic Digestion and Tandem Mass Spectrometry

The protein fractions were reduced with dithiothreitol (DTT), alkylated with iodoacetamide (IAA), and digested with MS grade trypsin (Pierce^TM^) before desalting with C18 ZipTip^®^ Pipette Tips. The tryptic peptides were then reconstituted in 7 µL of 0.1% formic acid in water and subjected to nano-electrospray ionization liquid chromatography–tandem mass spectrometry (nano-ESI-LCMS/MS). Peptide separation was performed by 1260 Infinity Nanoflow LC system (Agilent, Santa Clara, CA, USA) connected to Accurate-Mass Q-TOF 6550 series with a nanoelectrospray ionization source. The eluate was subjected to HPLC Large-Capacity Chip Column Zorbax 300-SB-C18 (160 nL enrichment column, 75 µm × 150 mm analytical column and 5 µm particles) (Agilent, Santa Clara, CA, USA). Injection volume was adjusted to 1 µL per sample, using a flow rate of 0.4 µL/min, with linear gradient of 5–70% of solvent B (0.1% formic acid in 100% acetonitrile). Drying gas flow was 11 L/min and drying gas temperature was 290 °C. Fragmentor voltage was 175 V, and the capillary voltage was set to 1800 V. Mass spectra were acquired using Mass Hunter acquisition software (Agilent, Santa Clara, CA, USA) in MS/MS mode with an MS scan range of 200–3000 *m*/*z* and MS/MS scan range of 50–3200 *m*/*z*. Data were extracted with MH+ mass range between 50 and 3200 Da and processed with Agilent Spectrum Mill MS Proteomics Workbench software packages version B.04.00 against a merged database incorporating both non-redundant NCBI databases of Serpentes (taxid: 8570) and an in-house transcripts database. Carbamidomethylation was specified as a fixed modification and oxidized methionine as a variable modification. The identified proteins or peptides were validated with the following filters: protein score > 5, and peptide score > 5. 

### 5.6. Estimation of Protein Relative Abundance

The relative protein abundances were estimated based on the chromatographic peak area of protein eluted and the mean spectral intensity (MSI) of peptides previously described [66]. The following calculation was adopted:

$$\mathrm{Relative}\;\mathrm{abundance}\;\mathrm{of}\;\mathrm{protein}\;X\;\mathrm{in}\;\mathrm{fraction}\;Y\;(\%)\;=\;\;\frac{\mathrm{MSI}\;\mathrm{of}\;\mathrm{protein}\;X\;\mathrm{in}\;\mathrm{HPLC}\;\mathrm{fraction}\;Y}{\mathrm{Total}\;\mathrm{MSI}\;\mathrm{of}\;\mathrm{all}\;\mathrm{proteins}\;\mathrm{in}\;\mathrm{HPLC}\;\mathrm{fraction}\;Y}\times \;\%\;\mathrm{Area}\;\mathrm{under}\;\mathrm{the}\;\mathrm{curve}\;(\mathrm{AUC})\;\mathrm{of}\;\mathrm{HPLC}\;\mathrm{fraction}\;Y$$

The MSI of protein X in fraction Y refers to the mean spectral intensity of the peptide ions assigned to protein X eluted in HPLC fraction Y. The fraction AUC was determined from the chromatogram using the Shimadzu LC Solution Software (Version 1.23, Shimadzu, Kyoto, Japan). 

### 5.7. Indirect Enzyme-Linked Immunosorbent Assay (ELISA) 

The immunological binding activity of NkMAV toward *N. sumatrana* venoms was performed using an indirect enzyme-linked immunosorbent assay (ELISA) with modification from the previous study [38]. *N. kaouthia* venom was used as the positive control. Briefly, 96-well immunoplates were coated with 10 ng venom antigen in 100 µL carbonate-bicarbonate coating buffer overnight at 4 °C. The plate was then flicked dry and rinsed three times with phosphate-buffered saline with 0.5% Tween^®^20 (PBST). Various dilutions of antivenom (1:150, 1:450, 1:1350, 1:4050, 1:12,150 and 1:36,450) from an antivenom stock concentration of 10 mg/mL were added to each antigen-coated well and incubated for an hour at room temperature. After washing the plate three times with PBST, 100 µL of appropriately diluted horseradish peroxidase-conjugated antihorse-IgG (Jackson ImmunoResearch Inc., West Grove, PA, USA) in PBST (1:10,000) was added to the well and incubated for another hour at room temperature. The excess unbound antibody-enzyme conjugate was removed by rinsing three times with PBST. Then, 100 µL of freshly prepared substrate solution (0.5 mg/mL o-phenylenediamine and 0.003% hydrogen peroxide in 0.1 M citrate-phosphate buffer, pH 5.0) was added to each well. The plate was left in dark for 30 min at room temperature, and the reaction was terminated by adding 50 µL of 12.5% sulfuric acid. The venom–antivenom complexes were monitored at 450 nm using an ELISA reader (SUNRISE-TECAN Type Touch Screen F039300, Tecan, Männedorf, Switzerland). The experiment was performed in triplicate, and absorbance values were expressed as means ± S.E.M. The half-maximal effective concentration (EC_50_) of venom-antivenom binding reaction was determined from the OD values through non-linear regression analysis using GraphPad Prism software (version 6.0, GraphPad Software Inc, La Jolla, CA, USA).

### 5.8. Determination of Venom Lethality and the Neutralization by Antivenom

The lethal activities of the *N. sumatrana* venoms were determined in ICR albino mice (n = 4 per dose, 20–25 g) as previously described [38]. Venoms of various doses of 100 µL were injected intravenously into the mice via caudal vein and the death ratios were recorded after 24 h. The median lethal doses (LD_50_) of the venoms were determined by Probit analysis, using the BioStat 2009 analysis software (AnalystSoft Inc., Vancouver, BC, Canada). LD_50_ was defined as the venom dose (µg/g) at which 50% of the mice were dead.

The neutralization study was performed by preincubating different doses of antivenom with a venom challenge dose of 2.5/5 LD_50_ at 37 °C for 30 min. The venom and antivenom mixtures were subsequently injected intravenously into the mice, and the survival ratios were recorded after 24 h. The median effective doses (ED_50_) of the venoms were determined by Probit analysis using BioStat 2009 analysis software (AnalystSoft Inc., Vancouver, Canada). ED_50_ was defined as the volume dose of antivenom (µL) at which 50% of mice survived. The neutralization efficacy was also expressed as median effective ratio (ER_50_), potency (P) and normalized potency (n-P) for comparative purposes [60,67].

## Figures and Tables

**Table 1:** Comparison of toxins identified in the *Naja sumatrana* venom proteomes sorted by protein families, subtypes, and relative abundances.

Protein Family/Protein Name	Database Accession ^a^	Relative Protein Abundance ^b^			
		Ns-NS (%) (32)	Ns-PG (%) (42)	Ns-TH (%) (22)	Ns-SU (%) (55)
**Three-finger toxins (3FTX)**		**58.58**	**63.86**	**69.82**	**57.71**
**Long neurotoxins (LNTX)**		**10.30**	**2.62**	**9.64**	**5.80**
Long neurotoxin 7	O42257	10.30	-	9.64	5.80
Long neurotoxin 3	P25671	-	0.52	-	-
Long neurotoxin 2	P25669	-	0.17	-	-
Long neurotoxin 7	O42257	-	1.94	-	-
**Short-neurotoxins (SNTX)**		**4.54**	**2.79**	**6.41**	**5.01**
Cobrotoxin homolog	Q9PTT0	3.13	0.42	-	-
Neurotoxin 3	Q9PSN6	1.40	0.79	4.05	2.94
Alpha-neurotoxin NTX-1	Q9YGJ6	-	-	-	0.53
Alpha-neurotoxin NTX-2	Q9YGJ5	-	-	1.48	0.50
Alpha-neurotoxin NTX-3	O57326	-	0.21	0.89	0.19
Neurotoxin homolog NL1	CL977.Contig3_NsM	-	1.37	-	-
Short neurotoxin 1	P60774	-	-	-	0.84
**Cytotoxin (CTX)**		**30.04**	**52.77**	**53.77**	**33.07**
Cytotoxin 2a	AAC61316	0.98	-	-	-
Cytotoxin 4N	Q9W6W9	0.84	1.30	14.70	-
Cytotoxin 2a	Q9PST4	5.11	-	-	-
Cytotoxin 6	P80245	-	-	3.99	-
Cytotoxin 5b	P60310	9.59	37.67	13.16	7.43
Cytotoxin 4	P60303	-	-	-	8.65
Cytotoxin 3	P60302	6.51	-	12.67	-
Cytotoxin 5	P07525	0.01	13.74	-	-
Cytotoxin 9	P01454	5.04	-	-	0.14
Cytotoxin 4	P01443	-	-	9.25	-
Cytotoxin 2	P01442	-	-	-	7.58
Cytotoxin 2	P01440	1.94	-	-	8.60
Cytotoxin 3	CL1512.Contig1_CiM	-	0.05	-	-
Cytotoxin 5	CL1026.Contig4_NkT	-	-	-	0.66
**Muscarinic toxin-like protein (MTLP)**		**-**	**2.02**	**-**	**9.60**
Muscarinic toxin-like protein	Q9W727	-	0.88	-	3.16
Muscarinic toxin-like protein 2	P82463	-	1.14	-	6.44
**Weak toxins (WTX)**		**13.71**	**3.66**	**-**	**4.22**
Weak neurotoxin 8	Q802B3	0.01	3.66	-	0.61
Weak toxin CM-9a	P25679	13.69	-	-	
Weak neurotoxin 6	P29180	-	-	-	2.97
Weak neurotoxin 6	O42256	-	-	-	0.65
**Phospholipase A2 (PLA2)**		**34.59**	**23.59**	**23.26**	**25.18**
Acidic phospholipase A2 D	Q9I900	10.12	0.75	3.12	8.58
Acidic phospholipase A2 C	Q92086	0.07	5.32	-	-
Neutral phospholipase A2 muscarinic inhibitor	Q92084	5.78	15.37	9.86	9.64
Acidic phospholipase A2 2	Q91133	6.97		-	-
Acidic phospholipase A2 DE-II	P00600	-	2.15	-	1.89
Acidic phospholipase A2 1	P00596	5.47	-	-	-
Acidic phospholipase A2 2	P00597	-	-	4.74	-
Acidic phospholipase A2 C	CL3683.Contig1_NsM	6.19	-	5.36	5.07
Neutral phospholipase A2 muscarinic inhibitor	CL2189.Contig1_CbM	-	-	0.19	-
**Venom nerve growth factor (vNGF)**		**2.79**	**6.63**	**0.87**	**3.21**
Venom nerve growth factor 2	Q5YF89	2.79	6.63	0.87	-
Venom nerve growth factor 2	CL429.Contig1_NnSL	-	-	-	3.21
**Kunitz-type serine protease inhibitor (KSPI)**		**-**	**-**	**-**	**5.86**
Kunitz-type serine protease inhibitor	CL534.Contig1_NkT	-	-	-	3.09
Kunitz-type serine protease inhibitor	unigene30356_NkT	-	-	-	2.45
Kunitz-type serine protease inhibitor	unigene8258_TwM	-	-	-	0.32
**Snake venom metalloproteinase (SVMP)**		**1.50**	**2.14**	**4.82**	**3.79**
Zinc metalloproteinase-disintegrin-like kaouthiagin-like	D3TTC1	0.75	-	1.76	-
Zinc metalloproteinase-disintegrin-like atragin	D3TTC2	-	0.89	1.77	-
Zinc metalloproteinase-disintegrin-like atrase-A	D5LMJ3	-	-	-	0.05
Zinc metalloproteinase-disintegrin-like cobrin	Q9PVK7	0.75	-	-	-
scutatease-1	B5KFV7	-	-	-	0.13
Zinc metalloproteinase-disintegrin-like kaouthiagin-like	CL626.Contig5_NsM2	-	0.17	-	0.39
Zinc metalloproteinase-disintegrin-like atragin	CL626.Contig4_NsM2	-	0.96	-	0.82
Zinc metalloproteinase-disintegrin-like atragin	CL626.Contig2_NsM2	-	-	-	0.45
Zinc metalloproteinase-disintegrin-like atragin	CL626.Contig1_NsM	-	-	1.29	0.92
Zinc metalloproteinase-disintegrin-like atragin	CL444.Contig1_NsM	-	0.13	-	0.42
Zinc metalloproteinase-disintegrin-like cobrin	CL115.Contig8_NkT	-	-	-	0.36
Snake venom metalloproteinase-disintegrin-like mocarhagin	CL115.Contig6_NkT	-	-	-	0.24
**Cobra venom factor (CVF)**		**1.18**	**1.39**	**0.35**	**1.36**
Cobra venom factor	Q91132	0.14	0.38	0.35	0.35
Complement C3	Q01833	-	-	-	0.20
Ophiophagus venom factor	I2C090	-	0.25	-	-
Cobra venom factor	CL4560.Contig1_NsM2	0.76	0.26	-	0.27
Cobra venom factor	CL1625.Contig1_NnSL	0.09	0.24	-	-
Cobra venom factor	CL472.Contig1_NkT	-	-	-	0.29
Cobra venom factor	Unigene370_NsM	0.19	0.26	-	0.24
**Phosphodiesterase (PDE)**		**0.74**	**0.89**	**0.63**	**0.46**
Venom phosphodiesterase	CL2341.Contig1_NsM	0.44	-	0.63	-
Venom phosphodiesterase	CL1403.Contig1_BcSL	0.07	-	-	-
Venom phosphodiesterase	CL1181.Contig1_NnSL	0.23	0.33	-	0.20
Venom phosphodiesterase	unigene5869_NsM	-	0.23	-	0.26
Venom phosphodiesterase	unigene19187_EsM	-	0.34	-	
**Cysteine-rich secretory protein (CRiSP)**		**0.39**	**0.17**	**-**	**0.47**
Cysteine-rich venom protein natrin-1	Q7T1K6	0.39	0.10	-	0.16
Cysteine-rich venom protein natrin-2	Q7ZZN8	-	-	-	0.09
Cysteine-rich venom protein natrin-1	CL2736.Contig1_NsM2	-	0.07	-	0.19
Cysteine-rich venom protein natrin-1	CL85.Contig1_NnSL	-	-	-	0.02
**L-amino acid oxidase (LAAO)**		**0.13**	**0.36**	**0.18**	**0.72**
L-amino acid oxidase	Q4JHE3	0.13	0.36	0.18	0.28
L-amino-acid oxidase	Q4JHE2	-	-	-	0.14
L-amino-acid oxidase	CL4047.Contig1_NsM2	-	-	-	0.30
**Snake venom serine protease (SVSP)**		**0.10**	**0.41**	**-**	**0.72**
Plasminogen activator	CL4067.Contig1_NsM	0.10	0.21	-	0.58
Serine protease harobin	unigene6087_NsM	-	0.19	-	-
Plasminogen activator	unigene33606_NnSL	-	-	-	0.14
**Hyaluronidase (HYA)**		**-**	**0.09**	**-**	**0.18**
Hyaluronidase	CL2939.Contig1_NkT	-	0.09	-	-
Hyaluronidase	unigene27829_NsM	-	-	-	0.18
**Vespryn**		**-**	**0.06**	**0.07**	**0.14**
Thaicobrin	P82885	-	0.04	0.07	0.01
Vesp22	F8RKW3	-	0.02	-	-
Ohanin	CL118.Contig3_NsM2	-	-	-	0.13
Ohanin	unigene31699_NnSL	-	0.00	-	-
**Acetylcholinesterase (AChE)**		**-**	**0.28**	**-**	**0.14**
Acetylcholinesterase	CL4231.Contig1_NsM	-	0.28	-	0.14
**5′ nucleotidase (5′NUC)**		**-**	**0.13**	**-**	**0.06**
Ecto-5′-nucleotidase 1a	A0A0F7YZM6	-	-	-	0.06
Snake venom 5′-nucleotidase	CL3600.Contig1_NsM2	-	0.13	-	-

Abbreviations: Ns-NS, Negeri Sembilan, Malaysia; Ns-PG, Penang, Malaysia; Ns-TH, Southern Thailand; Ns-SU, Sumatra, Indonesia. Protein identification and accession numbers were derived from databases based on best homology match, as shown in Supplementary Materials. ^a^ Accession numbers with suffix “_xxxx” were proteins identified based on tryptic peptides matched to sequences from an in-house transcript-database. ^b^ Relative abundance is represented as the percentage of total venom proteins, the value in parentheses indicates the number of proteoforms identified.

**Table 2:** Immunoreactivity of *Naja kaouthia* Monovalent Antivenom (NkMAV) toward *N. sumatrana* venoms from different geographical locales.

Venom	NkMAV	
	EC_50_ ^a^ (µg/mL)	Abs_max_ ^b^
*Naja sumatrana* (Negeri Sembilan)	9.64 ± 1.83	1.82 ± 0.15
*Naja sumatrana* (Penang)	8.20 ± 0.71	2.19 ± 0.12
*Naja sumatrana* (Southern Thailand)	6.34 ± 1.02	2.10 ± 0.11
*Naja sumatrana* (Sumatra)	4.06 ± 0.56	2.20 ± 0.07
*Naja kaouthia* (Thailand)	2.04 ± 0.32	2.40 ± 0.03

Values are expressed as mean ± S.E.M. of triplicates. NkMAV concentration = 42.99 ± 1.02 mg/mL. ^a^ EC_50_ (half-maximal effective concentration), defined as antivenom dose at which half-maximal binding occurred. ^b^ Abs_max_ (maximal absorbance), defined as maximal absorbance value at the highest concentration of antivenom.

**Table 3:** Lethality neutralization of *N. sumatrana* venoms from different geographical locales by NkMAV.

Venom	*i.v.* LD_50_ (µg/g)	Challenge Dose	ED_50_ (µL)	ER_50_ (mg/mL)	Potency, P (mg/mL)	Potency per Vial	Normalized Potency, n-P (mg/g)
Ns-NS	0.50 (0.40–0.62)	5 LD_50_	50.00	1.15 (0.93–1.43)	0.92	9.2	21.40
Ns-PG	0.96 (0.78–1.17)	2.5 LD_50_	36.96	1.49 (1.20–1.86)	0.90	9.0	20.94
Ns-TH	0.41 (0.36–0.47)	5 LD_50_	43.99	1.07 (0.97–1.18)	0.86	8.6	20.00
Ns-SU	0.39 (0.32–0.48)	5 LD_50_	73.93	0.61 (0.49–0.76)	0.49	4.9	11.40
*Naja kaouthia* (Thailand) *	0.18 (0.12–0.27)	5 LD_50_	18.75	1.15 (0.77–1.73)	0.92	9.2	20.44

Abbreviation: Ns-NS, *N. sumatrana* (Negeri Sembilan, Malaysia); Ns-PG, *N. sumatrana* (Penang, Malaysia); Ns-TH, *N. sumatrana* (Southern Thailand); Ns-SU, *N. sumatrana* (Sumatra, Indonesia). Note: LD_50_ (median lethal dose), dose of venom (µg/g) at which 50% of mice died. ED_50_ (median effective dose), dose of antivenom (µL) at which 50% of mice survived. ER_50_ (median effective ratio), ratio of venom (mg) to the volume does of antivenom (mL) at which 50% of mice survived. P (Potency), amount of venom (mg) completely neutralized per unit volume of antivenom (mL). n-P (normalized Potency), the neutralization potency of antivenom at which the amount of venom (mg) completely neutralized per unit amount of antivenom protein (g). Protein concentration of *Naja kaouthia* Monovalent Antivenom (NkMAV) estimated was 42.99 ± 1.02 mg/mL in this current study. Reconstituted antivenom had a volume of 10 mL per vial. * Values for *N. kaouthia* were adapted from Tan et al. [14,25], in which the NkMAV protein concentration was 45.0 ± 0.6 mg/g.

## Data Availability

The mass spectrometry proteomics data have been deposited into the ProteomeXchange Consortium (http://proteomecentral.proteomexchange.org) via the iProX partner repository [68] with the dataset identifier PXD034497 (released on 14 June 2022).

## Supplementary Materials

The following supporting information can be downloaded at: https://www.mdpi.com/article/10.3390/toxins14080522/s1, Table S1: Mass spectrometric data and peptide sequences of protein identified from the *Naja sumatrana* venom fractions using nano-ESI-LCMS/MS.

## References

[1] Gutiérrez J.M., Calvete J.J., Habib A.G., Harrison R.A., Williams D.J., Warrell D.A. (2017). Snakebite envenoming. Nat. Rev. Dis. Primers.

[2] Chippaux J.-P. (2017). Snakebite envenomation turns again into a neglected tropical disease!. J. Venom. Anim. Toxins Incl. Trop. Dis..

[3] Kasturiratne A., Wickremasinghe A.R., de Silva N., Gunawardena N.K., Pathmeswaran A., Premaratna R., Savioli L., Lalloo D.G., de Silva H.J. (2008). The global burden of snakebite: A literature analysis and modelling based on regional estimates of envenoming and deaths. PLoS Med..

[4] WHO (2016). Guidelines for the Management of Snakebites.

[5] Fry B.G. (2018). Snakebite: When the Human Touch Becomes a Bad Touch. Toxins.

[6] Uetz P., Hosek J. The Reptile Database. http://www.reptile-database.org/.

[7] Tan N.H., Tan K.Y., Tan C.H., Mackessy S.P. (2021). Snakebite in Southeast Asia: Envenomation and Clinical Management. Handbook of Venoms and Toxins of Reptiles.

[8] Wuster W. (1996). Taxonomic changes and toxinology: Systematic revisions of the Asiatic cobras (Naja naja species complex). Toxicon.

[9] Feola A., Marella G.L., Carfora A., Della Pietra B., Zangani P., Campobasso C.P. (2020). Snakebite Envenoming a Challenging Diagnosis for the Forensic Pathologist: A Systematic Review. Toxins.

[10] Kalita B., Patra A., Das A., Mukherjee A.K. (2018). Proteomic Analysis and Immuno-Profiling of Eastern India Russell’s Viper (*Daboia russelii*) Venom: Correlation between RVV Composition and Clinical Manifestations Post RV Bite. J. Proteome Res..

[11] Faisal T., Tan K.Y., Tan N.H., Sim S.M., Gnanathasan C.A., Tan C.H. (2021). Proteomics, toxicity and antivenom neutralization of Sri Lankan and Indian Russell’s viper (*Daboia russelii*) venoms. J. Venom. Anim. Toxins Incl. Trop. Dis..

[12] Pla D., Sanz L., Quesada-Bernat S., Villalta M., Baal J., Chowdhury M.A.W., Leon G., Gutierrez J.M., Kuch U., Calvete J.J. (2019). Phylovenomics of *Daboia russelii* across the Indian subcontinent. Bioactivities and comparative in vivo neutralization and in vitro third-generation antivenomics of antivenoms against venoms from India, Bangladesh and Sri Lanka. J. Proteom..

[13] Tan K.Y., Tan C.H., Sim S.M., Fung S.Y., Tan N.H. (2016). Geographical venom variations of the Southeast Asian monocled cobra (*Naja kaouthia*): Venom-induced neuromuscular depression and antivenom neutralization. Comp. Biochem. Physiology. Toxicol. Pharmacol..

[14] Tan K.Y., Tan C.H., Fung S.Y., Tan N.H. (2015). Venomics, lethality and neutralization of Naja kaouthia (*monocled cobra*) venoms from three different geographical regions of Southeast Asia. J. Proteom..

[15] Lingam T.M.C., Tan K.Y., Tan C.H. (2020). Proteomics and antivenom immunoprofiling of Russell’s viper (*Daboia siamensis*) venoms from Thailand and Indonesia. J. Venom. Anim. Toxins Incl. Trop. Dis..

[16] Hia Y.L., Tan K.Y., Tan C.H. (2020). Comparative venom proteomics of banded krait (*Bungarus fasciatus*) from five geographical locales: Correlation of venom lethality, immunoreactivity and antivenom neutralization. Acta Trop..

[17] Rusmili M.R.A., Othman I., Abidin S.A.Z., Yusof F.A., Ratanabanangkoon K., Chanhome L., Hodgson W.C., Chaisakul J. (2019). Variations in neurotoxicity and proteome profile of Malayan krait (*Bungarus candidus*) venoms. PLoS ONE.

[18] Tan C.H., Bourges A., Tan K.Y. (2022). King Cobra and snakebite envenomation: On the natural history, human-snake relationship and medical importance of Ophiophagus hannah. J. Venom. Anim. Toxins Incl. Trop. Dis..

[19] Tan K.Y., Ng T.S., Bourges A., Ismail A.K., Maharani T., Khomvilai S., Sitprija V., Tan N.H., Tan C.H. (2020). Geographical variations in king cobra (*Ophiophagus hannah*) venom from Thailand, Malaysia, Indonesia and China: On venom lethality, antivenom immunoreactivity and in vivo neutralization. Acta Trop..

[20] Tang E.L.H., Tan N.H., Fung S.Y., Tan C.H. (2019). Comparative proteomes, immunoreactivities and neutralization of procoagulant activities of *Calloselasma rhodostoma* (Malayan pit viper) venoms from four regions in Southeast Asia. Toxicon.

[21] Sivaganabalan R., Ismail A.K., Salleh M.S., Mohan K., Tan C.H., Adnan A., Ariff A.M., Mohamed Z., Thevarajah N., Daud R. (2017). Guideline: Management of Snakebite Ministry of Health Malaysia.

[22] Leong P.K., Sim S.M., Fung S.Y., Sumana K., Sitprija V., Tan N.H. (2012). Cross neutralization of Afro-Asian cobra and Asian krait venoms by a Thai polyvalent snake antivenom (Neuro Polyvalent Snake Antivenom). PLoS Negl. Trop. Dis..

[23] Leong P.K., Fung S.Y., Tan C.H., Sim S.M., Tan N.H. (2015). Immunological cross-reactivity and neutralization of the principal toxins of Naja sumatrana and related cobra venoms by a Thai polyvalent antivenom (Neuro Polyvalent Snake Antivenom). Acta Trop..

[24] Tan C.H. (2022). Snake Venomics: Fundamentals, Recent Updates, and a Look to the Next Decade. Toxins.

[25] Tan K.Y., Tan C.H., Fung S.Y., Tan N.H. (2016). Neutralization of the Principal Toxins from the Venoms of Thai Naja kaouthia and Malaysian Hydrophis schistosus: Insights into Toxin-Specific Neutralization by Two Different Antivenoms. Toxins.

[26] Ranawaka U.K., Lalloo D.G., de Silva H.J. (2013). Neurotoxicity in snakebite—The limits of our knowledge. PLoS Negl. Trop. Dis..

[27] Barber C.M., Isbister G.K., Hodgson W.C. (2013). Alpha neurotoxins. Toxicon.

[28] Tan C.H., Wong K.Y., Chong H.P., Tan N.H., Tan K.Y. (2019). Proteomic insights into short neurotoxin-driven, highly neurotoxic venom of Philippine cobra (*Naja philippinensis*) and toxicity correlation of cobra envenomation in Asia. J. Proteom..

[29] Ratanabanangkoon K., Tan K.Y., Eursakun S., Tan C.H., Simsiriwong P., Pamornsakda T., Wiriyarat W., Klinpayom C., Tan N.H. (2016). A Simple and Novel Strategy for the Production of a Pan-specific Antiserum against Elapid Snakes of Asia. PLoS Negl. Trop. Dis..

[30] Yap M.K., Fung S.Y., Tan K.Y., Tan N.H. (2014). Proteomic characterization of venom of the medically important Southeast Asian Naja sumatrana (Equatorial spitting cobra). Acta Trop..

[31] Chicheportiche R., Vincent J.P., Kopeyan C., Schweitz H., Lazdunski M. (1975). Structure-function relationship in the binding of snake neurotoxins to the torpedo membrane receptor. Biochemistry.

[32] Silva A., Cristofori-Armstrong B., Rash L.D., Hodgson W.C., Isbister G.K. (2018). Defining the role of post-synaptic α-neurotoxins in paralysis due to snake envenoming in humans. Cell. Mol. Life Sci..

[33] Shan L.L., Gao J.F., Zhang Y.X., Shen S.S., He Y., Wang J., Ma X.M., Ji X. (2016). Proteomic characterization and comparison of venoms from two elapid snakes (*Bungarus multicinctus* and *Naja atra*) from China. J. Proteom..

[34] Tan N.H., Wong K.Y., Tan C.H. (2017). Venomics of Naja sputatrix, the Javan spitting cobra: A short neurotoxin-driven venom needing improved antivenom neutralization. J. Proteom..

[35] Palasuberniam P., Chan Y.W., Tan K.Y., Tan C.H. (2021). Snake Venom Proteomics of Samar Cobra (*Naja samarensis*) from the Southern Philippines: Short Alpha-Neurotoxins as the Dominant Lethal Component Weakly Cross-Neutralized by the Philippine Cobra Antivenom. Front. Pharmacol..

[36] Wong K.Y., Tan C.H., Tan N.H. (2016). Venom and Purified Toxins of the Spectacled Cobra (*Naja naja*) from Pakistan: Insights into Toxicity and Antivenom Neutralization. Am. J. Trop. Med. Hyg..

[37] Tan C.H., Tan K.Y., Ng T.S., Sim S.M., Tan N.H. (2018). Venom Proteome of Spine-Bellied Sea Snake (*Hydrophis curtus*) from Penang, Malaysia: Toxicity Correlation, Immunoprofiling and Cross-Neutralization by Sea Snake Antivenom. Toxins.

[38] Tan C.H., Wong K.Y., Tan K.Y., Tan N.H. (2017). Venom proteome of the yellow-lipped sea krait, Laticauda colubrina from Bali: Insights into subvenomic diversity, venom antigenicity and cross-neutralization by antivenom. J. Proteom..

[39] Chong H.P., Tan K.Y., Tan N.H., Tan C.H. (2019). Exploring the diversity and novelty of toxin genes in *Naja sumatrana*, the Equatorial spitting cobra from Malaysia through de novo venom-gland transcriptomics. Toxins.

[40] Tan K.Y., Tan C.H., Chanhome L., Tan N.H. (2017). Comparative venom gland transcriptomics of Naja kaouthia (*monocled cobra*) from Malaysia and Thailand: Elucidating geographical venom variation and insights into sequence novelty. PeerJ.

[41] Pruksaphon K., Yuvaniyama J., Ratanabanangkoon K. (2022). Immunogenicity of snake α-neurotoxins and the CD4 T cell epitopes. Toxicon.

[42] Wong K.Y., Tan K.Y., Tan N.H., Tan C.H. (2021). A Neurotoxic Snake Venom without Phospholipase A2: Proteomics and Cross-Neutralization of the Venom from Senegalese Cobra, Naja senegalensis (Subgenus: Uraeus). Toxins.

[43] Petras D., Sanz L., Segura A., Herrera M., Villalta M., Solano D., Vargas M., Leon G., Warrell D.A., Theakston R.D. (2011). Snake venomics of African spitting cobras: Toxin composition and assessment of congeneric cross-reactivity of the pan-African EchiTAb-Plus-ICP antivenom by antivenomics and neutralization approaches. J. Proteome Res..

[44] Wong K.Y., Tan C.H., Tan K.Y., Quraishi N.H., Tan N.H. (2018). Elucidating the biogeographical variation of the venom of Naja naja (*spectacled cobra*) from Pakistan through a venom-decomplexing proteomic study. J. Proteom..

[45] Senji Laxme R.R., Attarde S., Khochare S., Suranse V., Martin G., Casewell N.R., Whitaker R., Sunagar K. (2021). Biogeographical venom variation in the Indian spectacled cobra (*Naja naja*) underscores the pressing need for pan-India efficacious snakebite therapy. PLoS Negl. Trop. Dis..

[46] Dutta S., Chanda A., Kalita B., Islam T., Patra A., Mukherjee A.K. (2017). Proteomic analysis to unravel the complex venom proteome of eastern India Naja naja: Correlation of venom composition with its biochemical and pharmacological properties. J. Proteom..

[47] Tan K.Y., Wong K.Y., Tan N.H., Tan C.H. (2020). Quantitative proteomics of Naja annulifera (sub-Saharan snouted cobra) venom and neutralization activities of two antivenoms in Africa. Int. J. Biol. Macromol..

[48] Huang H.W., Liu B.S., Chien K.Y., Chiang L.C., Huang S.Y., Sung W.C., Wu W.G. (2015). Cobra venom proteome and glycome determined from individual snakes of Naja atra reveal medically important dynamic range and systematic geographic variation. J. Proteom..

[49] Chong H.P., Tan K.Y., Liu B.S., Sung W.C., Tan C.H. (2022). Cytotoxicity of Venoms and Cytotoxins from Asiatic Cobras (*Naja kaouthia*, *Naja sumatrana*, *Naja atra*) and Neutralization by Antivenoms from Thailand, Vietnam, and Taiwan. Toxins.

[50] Liu C.C., Chou Y.S., Chen C.Y., Liu K.L., Huang G.J., Yu J.S., Wu C.J., Liaw G.W., Hsieh C.H., Chen C.K. (2020). Pathogenesis of local necrosis induced by Naja atra venom: Assessment of the neutralization ability of Taiwanese freeze-dried neurotoxic antivenom in animal models. PLoS Negl. Trop. Dis..

[51] Panagides N., Jackson T.N., Ikonomopoulou M.P., Arbuckle K., Pretzler R., Yang D.C., Ali S.A., Koludarov I., Dobson J., Sanker B. (2017). How the Cobra Got Its Flesh-Eating Venom: Cytotoxicity as a Defensive Innovation and Its Co-Evolution with Hooding, Aposematic Marking, and Spitting. Toxins.

[52] Chong H.P., Tan K.Y., Tan C.H. (2020). Cytotoxicity of Snake Venoms and Cytotoxins From Two Southeast Asian Cobras (*Naja sumatrana*, *Naja kaouthia*): Exploration of Anticancer Potential, Selectivity, and Cell Death Mechanism. Front. Mol. Biosci..

[53] Gutiérrez J.M., Lomonte B. (2013). Phospholipases A2: Unveiling the secrets of a functionally versatile group of snake venom toxins. Toxicon.

[54] Tan C.H., Wong K.Y., Tan N.H., Ng T.S., Tan K.Y. (2019). Distinctive Distribution of Secretory Phospholipases A₂ in the Venoms of Afro-Asian Cobras (*Subgenus: Naja*, *Afronaja*, *Boulengerina* and *Uraeus*). Toxins.

[55] Tan C.H., Tan K.Y., Lim S.E., Tan N.H. (2015). Venomics of the beaked sea snake, Hydrophis schistosus: A minimalist toxin arsenal and its cross-neutralization by heterologous antivenoms. J. Proteom..

[56] Yamazaki Y., Morita T. (2004). Structure and function of snake venom cysteine-rich secretory proteins. Toxicon.

[57] Vogel C.W., Fritzinger D.C. (2010). Cobra venom factor: Structure, function, and humanization for therapeutic complement depletion. Toxicon.

[58] Dhananjaya B.L., CJ D.S. (2010). An overview on nucleases (DNase, RNase, and phosphodiesterase) in snake venoms. Biochem. Biokhimiia.

[59] Silva-de-França F., Villas-Boas I.M., Serrano S.M.d.T., Cogliati B., Chudzinski S.A.d.A., Lopes P.H., Kitano E.S., Okamoto C.K., Tambourgi D.V. (2019). Naja annulifera Snake: New insights into the venom components and pathogenesis of envenomation. PLoS Negl.Trop. Dis..

[60] Tan C.H., Tan K.Y. (2019). Functional Application of Snake Venom Proteomics in In Vivo Antivenom Assessment. Methods Mol. Biol..

[61] WHO (2010). WHO Guidelines for the Production, Control and Regulation of Snake Antivenom Immunoglobulins.

[62] Ratanabanangkoon K. (2021). A Quest for a Universal Plasma-Derived Antivenom Against All Elapid Neurotoxic Snake Venoms. Front. Immunol..

[63] Ratanabanangkoon K., Tan K.Y., Pruksaphon K., Klinpayom C., Gutiérrez J.M., Quraishi N.H., Tan C.H. (2020). A pan-specific antiserum produced by a novel immunization strategy shows a high spectrum of neutralization against neurotoxic snake venoms. Sci. Rep..

[64] Nair A.B., Jacob S. (2016). A simple practice guide for dose conversion between animals and human. J. Basic Clin. Pharm..

[65] Tongpoo A., Trakulsrichai S., Putichote K., Sriapha C., Wananukul W. (2019). Recurrent neurotoxic envenoming of cobra bite. Toxicon.

[66] Tan C.H., Tan K.Y., Tan N.H. (2019). A Protein Decomplexation Strategy in Snake Venom Proteomics. Methods Mol. Biol..

[67] Araujo H.P., Bourguignon S.C., Boller M.A., Dias A.A., Lucas E.P., Santos I.C., Delgado I.F. (2008). Potency evaluation of antivenoms in Brazil: The national control laboratory experience between 2000 and 2006. Toxicon.

[68] Ma J., Chen T., Wu S., Yang C., Bai M., Shu K., Li K., Zhang G., Jin Z., He F. (2019). iProX: An integrated proteome resource. Nucleic Acids Res..

